# Determination of the Location and Magnetic Moment of Ferromagnetic Objects Based on the Analysis of Magnetovision Measurements

**DOI:** 10.3390/s19020337

**Published:** 2019-01-16

**Authors:** Michał Nowicki, Roman Szewczyk

**Affiliations:** Warsaw University of Technology, Institute of Metrology and Biomedical Engineering, 02-525 Warsaw, Poland; szewczyk@mchtr.pw.edu.pl

**Keywords:** magnetovision, objects detection, magnetic anomalies

## Abstract

This article is concerned with the localization of ferromagnetic objects on the basis of magnetovision measurement analysis. In the presented case, the concept of localization is understood as the indication of the *x*, *y*, and *z* coordinates of the magnetic moment of the sought object. Magnetovision measurement provides a much simpler, two-dimensional localization of magnetic anomalies compared to existing active and passive mobile devices, largely based on operator knowledge and experience. In addition, the analysis of the obtained magnetovision measurement, by fusing data with a mathematical model, enables a quantitative assessment of the position of an object in space and the determination of the value and spatial orientation of its magnetic moment vector. The detection and localization method was verified using the certified magnetic moment standard. An additional novelty is the inclusion of the influence of the constant gradient of the external field in the model, which corresponds to disturbing the measurement by the influence of large, but distant, objects. The proposed three-dimensional magnetovision measurement method and its analysis enable the determination of the x, y, and z coordinates; the angular position; and the magnetic moment values of unknown magnetic dipoles in real conditions (effects of disturbances generated by other distant objects and background noise), thus precisely detecting and locating the ferromagnetic object.

## 1. Introduction

There is an ongoing need to develop quantitative methods of hidden objects detection, mainly in the field of humanitarian demining. ERW (Explosive Remnants of War), landmines, and IEDs are a threat to both the population and economy of affected regions. 

ERWs are often found at depths outside the range of conventional, active metal detectors [1]. This may be due to natural soil-forming phenomena, but is more often due to the way in which the mine or unexploded ordnance found itself in a given place. For objects that strongly hit the ground, significant depths are recorded, even up to 3 m. Examples are mortar shells, artillery shells, and aerial bombs. The percentage of unexploded ordnance is estimated to be 15% [1]. 

The sensitivity of the active metal detector drops exponentially with the distance of the object from the detector coils. The working range of the detector is usually 2 to 3 coil diameters. Most conventional active metal detectors used in demining have a working range below 20 cm [2]. 

Deeply located unexploded ordnance does not pose a direct threat to pedestrians, but threatens heavy machinery, construction, and agricultural activities in a given area. Therefore, after the standard demining, a second long-range test is often used (Deep Search). Two main methods are used for such research: active and passive [3]. 

Active devices have the same principle of operation as metal detectors used in conventional de-mining, but have much larger transmitting and receiving coils, which are about 1 m in diameter. Increasing the diameter proportionally increases the detector’s range, so for a 1 m diameter, it will be about 3 m, but at the same time, reduces the detector’s *XY* resolution and sensitivity to small objects that can remain undetected. The biggest advantage of active technologies is their ability to detect all metals and other conductive objects. Unfortunately, as with all active techniques, they are prone to anti-engineering traps [4].

Passive instruments are sensitive magnetometers that measure the scalar or vector value of the natural Earth’s magnetic field [5]. The presence of a ferromagnetic object, such as an air bomb with a steel body, causes a disturbance of the natural magnetic field [6]. Because these instruments are based on measuring the value, distribution, or gradient of a constant magnetic field, they can only detect ferromagnetic objects. Currently, the operation of such mobile devices consists of performing detailed measurements using a hand-held magnetometer in a given area, and determining the location and depth of the object on the basis of the shape of the local magnetic field disturbance, i.e., anomaly. Devices of this type detect large objects at depths of up to 5 m [7]. The most common are fluxgate sensors, due to their high resolution of up to 100 pT [8]. The most effective way of detecting deeply located objects is the use of geo-mapping technology, which allows the archiving and analysis of measurement data and minimizes human error [9]. Currently used systems rely on terrain scanning through ’manual’ transport of a magnetometer coupled with a GPS receiver. Suitable bogies or vehicles are also used, for which the disturbance generated by the vehicle should be taken into account [10].

Much effort has been devoted to developing methods for detecting and identifying objects on the basis of libraries of expected magnetic signatures [11]. These libraries are most often created on the basis of dipole analytical models [12]. Attempts to discriminate objects often consist of measuring the mutual scale of the remanence effect and the moment induced by the Earth’s natural field [13]. Preliminary results indicate the possibility of distinguishing such munitions from other accidental ferromagnetic objects [14]. The attempt to introduce a metrological approach, i.e., to determine uncertainty in the detection and location of unexploded ordnance, is presented in the paper [15].

On a larger scale, passive methods are used for the detection of shipwrecks [16] and submarines [17], and for the prospecting of resources [18,19]. Various analytical methods of data inversion for the calculation of magnetic dipole location [20,21,22,23,24,25,26,27] are known, and there is ongoing progress in the number of unknown variables which can by calculated, as well as the calculation time [28].

Nonetheless, there is still a need for progress in fast, automatic, quantitative methods of interpreting data from passive systems, and in the overall measurement accuracy.

A relatively new passive method is magnetovision. Initially, the magnetovision systems consisted of a scanning system and a magnetoresistance sensor, enabling the measurement of the magnetic field distribution. Their most prominent features are the amount of gathered data, with a high spatial resolution, and a high accuracy of measurement points coordinates [29]. 

Initially, magnetovision was associated in the literature with measurements of the magnetic properties of electrical steel, with an attempt to link the magnetism measurement with the grain system in the samples [30,31]. Later versions of the system were used to measure the distribution of the field in open waveguides, and to verify numerical calculations [32]. Contemporary systems are composed of magnetometers matrices, allowing for real-time visualization of the magnetic field distribution [33].

With proper analysis, the magnetic moment of the object can be deduced from the magnetovision pattern. The magnetic moment is the magnitude determining the shape and size of the magnetic anomaly, i.e., the distribution of the induction of the magnetic field in the space around the object. The value of induction of the magnetic field **B** in a plane perpendicular to a given vector of magnetic moment, assuming an appropriately large distance, can be calculated from well-known formulas of potential field theory, e.g., [34]. 

Furthermore, it may be promising to use the presented approach to, e.g., locate shipwrecks using datasets from large marine areas [35], or locate hidden ferromagnetic bodies, both in medical [26] and industrial conditions [36]. The presented approach could also be combined with recent advances in magnetic tracking systems, allowing, e.g., for particle tracking in dense fluidized beds [37], or in motion-tracking systems [38].

## 2. Materials and Methods

Because many dangerous objects, such as unexploded ordnance, IEDs, or mines, are made of ferromagnetic steel, they have distinct ferromagnetic properties. High magnetic permeability makes them magnetized in the Earth’s field, and they have their own magnetic remanence, which is dependent on their magnetic history, such as the production process and possible shocks. 

The most important of the samples used is the ME 8 magnetic moment standard [39], consisting of high-stability permanent magnets produced by Magnet-Physik Dr Steingroever GmbH (Köln, Germany), equipped with appropriate calibration certificates. The sample was a cuboid approximately 10 mm × 16 mm × 17 mm, sunk in protective resin. As it is made of a hard magnetic material with a very low temperature coefficient, its magnetic signatures do not depend on external, weak magnetic fields, such as the Earth’s magnetic field. The use of the standard and its precise positioning allow us to relate the results of the analysis of magnetovision measurements to real conditions, thus the results obtained are quantitative, not qualitative, which is a novelty of this work in relation to the current research on the detection of ferromagnetic objects.

The magnetic moment value of the ME8 standard is m = 0.603 Am^2^.

**Figure 1:**
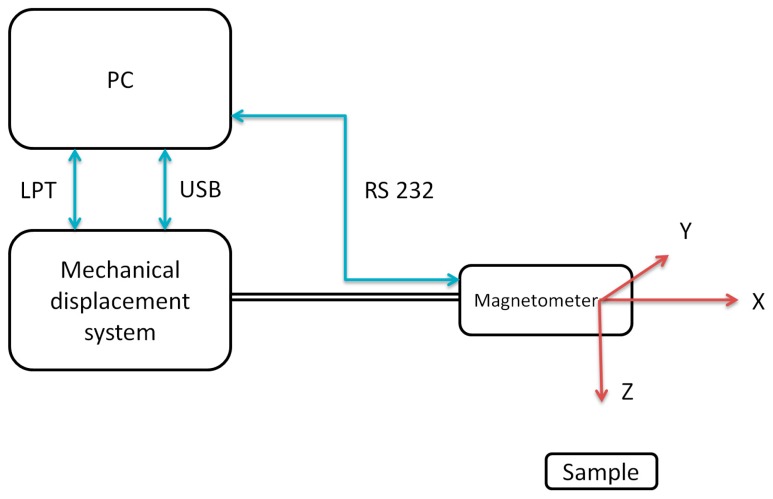
Schematic block diagram of the magnetovision scanning system.

Magnetic field distribution tests, in order to obtain magnetovision images, were carried out using the developed test stand, the diagram of which is shown in the Figure 1.

A PC computer with a specially prepared application in the LabView environment is responsible for controlling the entire measurement system and acquiring data from the sensor. The system works with the scanner system by means of the LPT and USB interface, while the sensor is equipped with a magnetoresistance sensor using the RS232 serial interface. The description of the measurement stand is provided in [40]. 

The developed station uses a Honeywell HMR 2300 precision three-axial magnetoresistance sensor. This sensor enables the measurement of magnetic induction in three axes, with a resolution of 7 nT. This is a significant advantage over measurements made with uniaxial sensors, because it allows the value and direction of the magnetic induction vector at a given measurement point to be determined.

The magnetoresistive sensor was placed at the extremity of the arm of the mechanical system, allowing the sensor to be moved along parallel lines (with a given pitch), indicating the measurement plane. The uncertainty of the positioning of the measuring points was estimated at 1 mm. During the measurements, no additional magnetizing fields were used, and only background disturbances were measured, which are mainly the Earth’s natural magnetic field. An area of 200 mm × 200 mm was assumed for the tests, on which 11 parallel measuring lines were determined. There are 100 to 1000 measurement points on each line. The mentioned parameters were selected depending on the desired resolution and measurement time. 

Obtained results were processed in the Matlab program, assigning them to individual measurement lines. Then, the results of the obtained matrix were interpolated to the required number of points, which allowed us to obtain a magnetovision image. During the measurement, data from the *XY* plane were collected at a fixed *Z* height. The station makes it possible to move the measurement plane in the *Z* axis by the given value. This makes it possible to obtain magnetovision images at different heights above the sample. The use of a second magnetometer in the scanner system, shifted in the *Z* axis by a given value, allows simultaneous measurement at two heights or a measurement of the field gradient.

**Figure 2:**
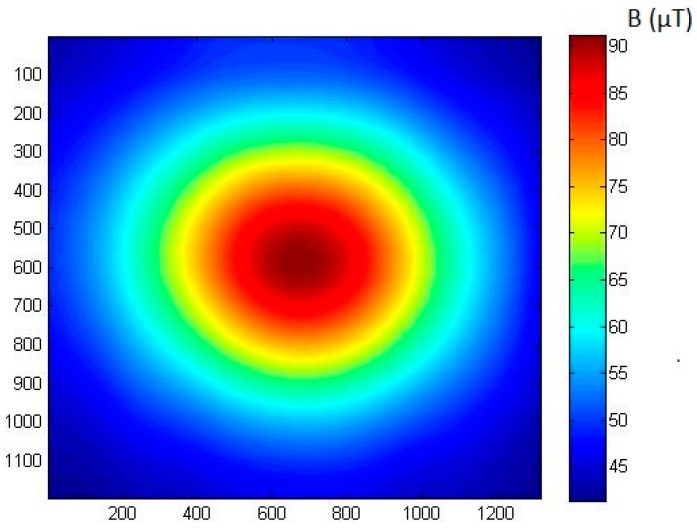
Sample magnetovision measurement of magnetic field induction of ME8 magnetic moment standard. Coordinates of the plot are *X* and *Y* (horizontal measurement plane), and vertical component *B_Z_* of the magnetic field is shown. The axis of the standard was perpendicular to the measurement plane, and the vertical distance *z*_0_ was set to 140 mm.

The inconvenience of earlier solutions known from the literature is solved by the method described in this paper, allowing for the simultaneous determination of all coordinates (*x*, *y*, *z*) of the searched object and the value and direction of its resultant magnetic moment. This method consists of analyzing the distribution of the induction value B of the magnetic field in the measuring plane above the object. Thus, it is the development of the basic magnetovision measurement that only visualized this distribution. Figure 2 shows the geometric scheme of the measurement plane and hidden object, as well as the adopted designations of individual parameters.

Center 0 of the local *XYZ* coordinate system is located in the center of the magnetovision measurement plane. The assumed symbols: *x*_0_, *y*_0_, *z*_0_—the coordinates of the center of the searched object in relation to the center of the local coordinate system; ***m***—the resultant magnetic moment of the object; ***m_XY_***—projection of the moment vector into a plane *XY*; *φ*—the angle between the vector of the moment ***m*** and the vertical axis *Z*; and *β*—angle between the *X* axis and the vector ***m_XY_***.

**Figure 3:**
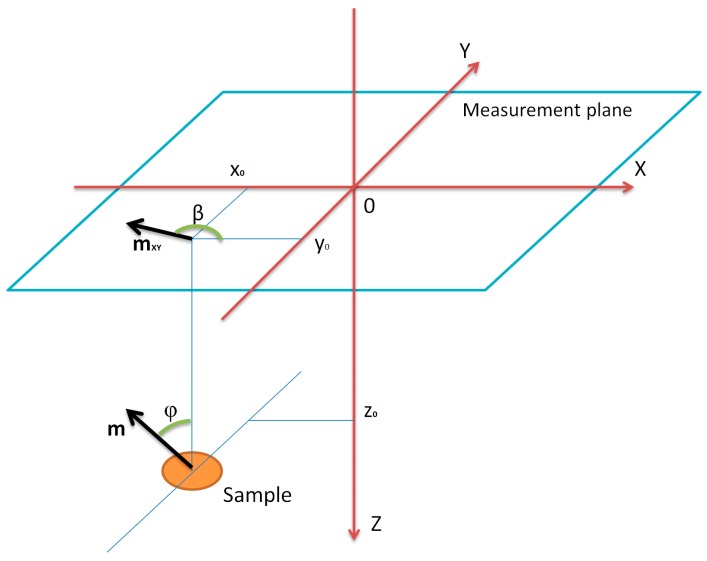
Measurement plane and sample geometrical values.

On the basis of the basic dipole dependences known from the literature [34], the dependence of the vertical component of induction ***B*** of the magnetic field at individual measurement points on the plane at an unknown distance *z*_0_ from the searched object with the resultant magnetic moment ***m*** can be written. The detailed derivation of the analogous formula is presented in [41].

(1) $$\begin{array}{cc} B_{z} & =\;\frac{0.3\;m\sin\left(\varphi \right)\cos\left(\beta \right)\left(x-x_{0}\right)z_{0}}{{\left({\left(x-x_{0}\right)}^{2}+{\left(y-y_{0}\right)}^{2}+z^{2}\right)}^{\frac{5}{2}}}\\ & +\;\frac{0.3\;m\sin\left(\varphi \right)\sin\left(\beta \right)\left(y-y_{0}\right)z_{0}}{{\left({\left(x-x_{0}\right)}^{2}+{\left(y-y_{0}\right)}^{2}+z^{2}\right)}^{\frac{5}{2}}}\\ & +\;\frac{0.1\;m\cos\left(\varphi \right)\left(2z_{o}^{2}-{\left(x-x_{0}\right)}^{2}-{\left(y-y_{0}\right)}^{2}\right)}{{\left({\left(x-x_{0}\right)}^{2}+{\left(y-y_{0}\right)}^{2}-z^{2}\right)}^{\frac{5}{2}}}\;+c+dx\\ & +ey \end{array}$$

where the known values are:

*B_z_*—the value of the magnetic field induction component in the *Z* (‘vertical’) axis for a given point on the *XY* plane in µT;

*x*, *y*, *z*—coordinates of the center measurement point relative to the center of the local coordinate system in meters,

and the unknown are:

$x_{0}$, $y_{0}$, $z_{0}$—coordinates of the center of the object sought relative to the center of the local coordinate system in meters, accordingly to Figure 3; 

*m*—value of the resultant magnetic moment of the object in Am^2^; 

*φ*—the angle between the moment vector and the vertical *Z* axis; 

*β*—the angle between the *X* axis and the projection of the moment vector on the *XY* plane;

*c*—the vertical component of the local background field in µT; 

*d,e*—substitute gradient coefficients, allowing the model to be adjusted in non-homogeneous background field conditions, in µT/m. 

The unknown values are denoted with subscript x_0_ for values set by the experimenter, and with subscript $x_{w}$ obtained from the inverse problem calculation.

Equation (1) determines the distribution of induction *B_z_* of the magnetic field in the plane at a distance $z_{0}$ from the object with the moment ***m*** and the coordinates $x_{0}$, $y_{0}$. In addition, the use of coefficients c, d, and e allows the influence of a non-homogeneous background field, i.e., the terrestrial field and gradients from large, significantly distant objects, to be taken into account.

Using Equation (1) or analogous equations [36], it is possible to determine the distribution of the value of field B, knowing all the parameters appearing on the right side of the Equation (1), and similar examples are known from the literature. However, the reverse problem can be solved—on the basis of measuring the value of magnetic field induction *B_z_* in points with known *x, y* coordinates, all parameters determining the resultant magnetic moment of an unknown, hidden ferromagnetic object can be identified. These are: calculated coordinates $x_{w}$, $y_{w}$, $z_{w}$ of the center of the resultant magnetic moment; the value of the magnetic moment *m_w_*; and angles *φ_w_* and *β_w_* defining the direction of the vector of magnetic moment.

The measurement data were fitted to the model (1) in Matlab’s Curve Fitting Toolbox, with the Levenberg-Marquardt algorithm for non-linear least squares fit execution. It was found that additional LAR (Least Absolute Residual) preprocessing increases the robustness of the fitting procedure. The LAR method finds a curve that minimizes the absolute difference of the residuals. Therefore, outliers have a lesser influence on the fit. Data was also preprocessed with a moving average digital filter for noise suppression. Other default possibilities, such as the standard non-linear least squares algorithm or bi-square preprocessing, were stuck in local minima, and could not cope with all of Equation (1)’s variables. Thus, with readily available 2D curve fit functionality, it was possible to determine the model parameters, including the coordinates and magnetic moment value of the tested object.

The presented equation and selection of commercial, user-friendly curve fitting algorithms allow for fast inverse dipole problem solving, which may be utilized, e.g., for ferromagnetic objects location. Many achievements in this area have been presented already, with an increasing number of unknown variables, e.g., Munschy et al. presented inversion which can be performed on any dataset with six unknown variables—the magnetic moment coordinates and their three moment vectors [42]. The geomagnetic field, however, was set as known, and there was no compensation for field inhomogeneity. The presented model (1) allows for the determination of nine variables—magnetic moment coordinates and their orientation (by its value and two angles), and the external constant field and its two gradients.

## 3. Results

**Figure 4:**
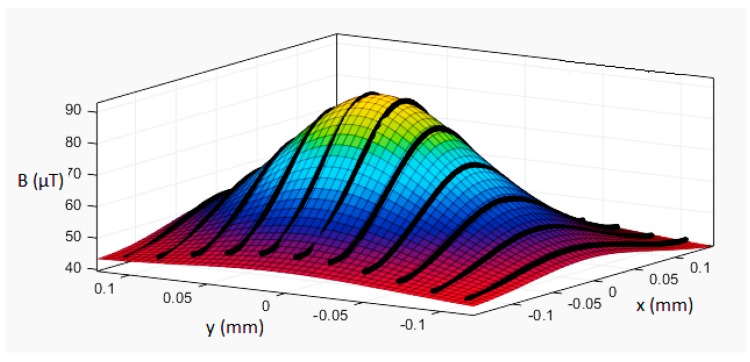
Approximation of ME8 induction of magnetic field distribution (black lines—measurement results) to the model (4). Coordinates set: (*x*_0_, *y*_0_, *z*_0_) = (0, 0, 140) mm, *β* = 0°, and *φ* = 0°. Three-dimensional graph of the distribution of *B* field values.

**Figure 5:**
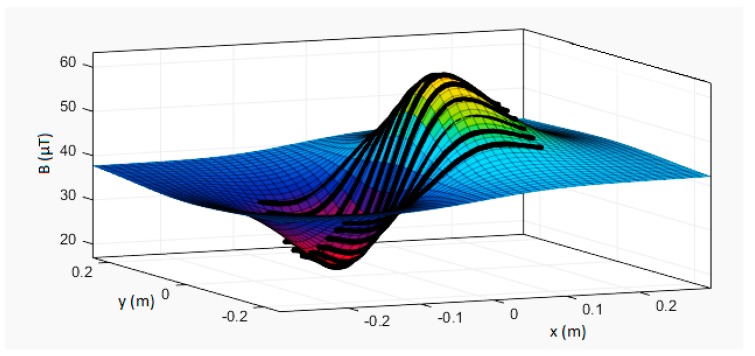
Approximation of ME8 induction of magnetic field distribution (black lines—measurement results) to the model (4). Coordinates set: (*x*_0_, *y*_0_, *z*_0_) = (0, 0, 140) mm, *β* = 0°, and *φ* = 90°. Three-dimensional graph of the distribution of *B* field values. The plane of the background field is inclined due to the gradient of the magnetic field—the heterogeneity of the background field.

In order to check the operation of the model (1), a series of ME8 magnetic moment standard measurements were carried out. In the first series of measurements, the parameters in Equation (1) were not changed, i.e., the reproducibility and spread of the results were checked. A series of magnetovision measurements were made with the following parameters: coordinates *x*_0_ = 30 mm, *y*_0_ = −30 mm, *z*_0_ = 160 mm, angle *β* = 0°, and angle *φ* = 65°. The obtained results are presented in Table 1. Exemplary comparison of model and measurement results is presented in Figure 4 and Figure 5. 

The method allowed us to determine the distance from the standard with an uncertainty of 0.5%. A high accuracy was also obtained for the measurement of the value of the background field and the angles *φ, β*. Significantly higher uncertainty occurs when measuring the *x, y* coordinates and values of the magnetic moment m. However, it should be emphasized that the calculated center of the object (*x_w_, y_w_, z_w_*) is inside its contour. Additionally, the measurement of the magnetic moment is made with a greater accuracy than with other non-laboratory methods used in practice, and with all parameters of the moment vector assumed as unknown.

In the next series of measurements, the standard was placed on a rotary table, enabling us to change the angle *φ* with respect to the measuring plane. A series of magnetovision measurements were made with the following parameters: coordinates *x*_0_ = 0, *y*_0_ = 0, *z*_0_ = 140 mm, angle *β* = 0°, and angle *φ* changed from 0 to 90° with a resolution of 5°. 

**Figure 6:**
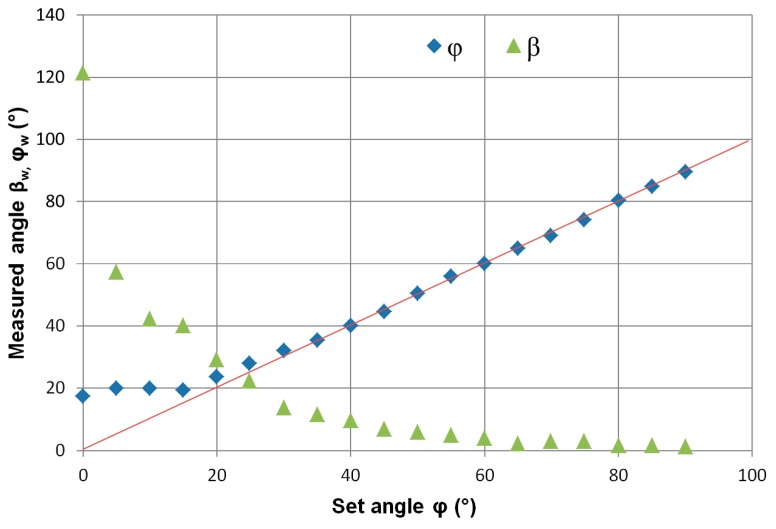
Dependence of the determined values of angles *φ_w_* and *β_w_* on the set angle *φ*. Set ME8 standard coordinates (*x*_0_, *y*_0_, *z*_0_) = (0, 0, 140) mm.

The dependence of determining the angles *β* and φ for the set angle *φ* is more complex, and it is shown in Figure 6. The graph shows that the determination of the angle *φ* is more accurate and only has a significant error in the range of 0–20°. Determining the *β* angle, however, is more difficult, because the measured value tends asymptotically to the set point in the whole range of angle change *φ*. The analysis of the geometrical system indicates that for small angles *φ*, the influence of the value of the angle *β* on the fit of the model is minimal, hence the great uncertainty of its determination. At the same time, for small values of the angle *φ*, determination of the angle *β* with high uncertainty does not significantly influence the determination of the value of magnetic moment m_w_ and spatial coordinates *x_w_, y_w_, z_w_*.

**Figure 7:**
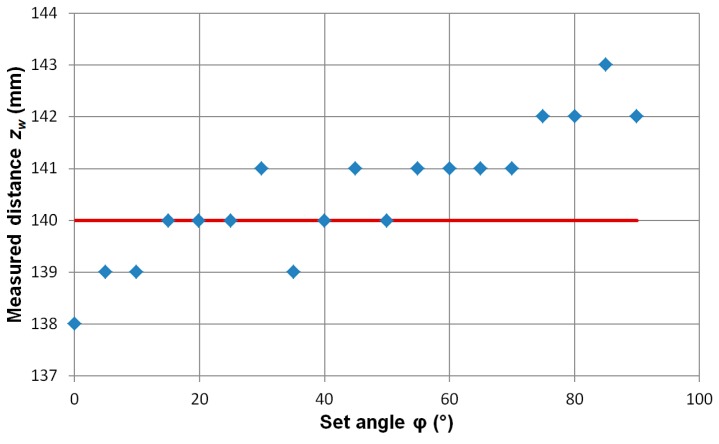
Dependence of the determined distance value from the object on the set angle *φ*. Set coordinates of the ME8 standard (*x*_0_*, y*_0_*, z*_0_) = (0, 0, 140) mm. Red line—the set value *z*_0_ of the object.

**Figure 8:**
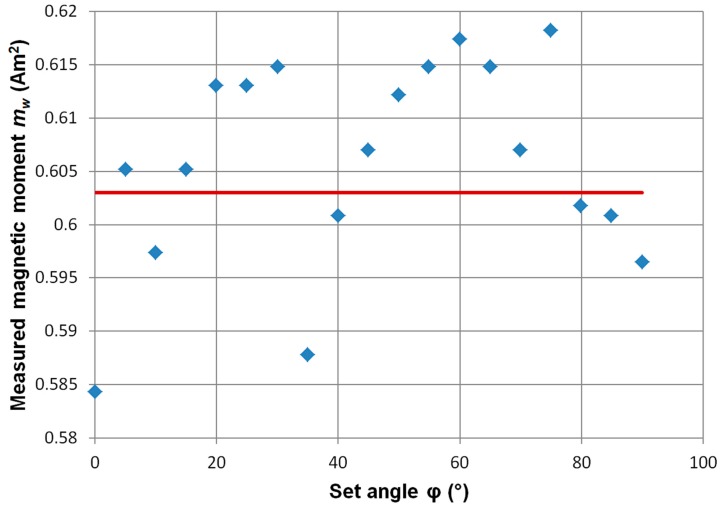
Dependence of the determined magnetic moment value of the object on the set angle *φ*. Set coordinates of the ME8 standard (*x*_0_, *y*_0_, *z*_0_) = (0, 0, 140) mm. Red line—the *m* value of the object.

Figure 7 is a graph of the dependence of changes in the measured distance from the object at different angles *φ*. The angle *φ* does not have a distinct influence on the calculated distance from the hidden ferromagnetic object. An analogous situation occurs for the measurement of magnetic moment (Figure 8). Here too, the influence of the angle *φ* is negligible compared to the uncertainty of the measurement.

The most important finding in the results of these tests is that the direction of the vector of the magnetic moment of the searched object relative to the measuring plane (angles *β, φ*) can be arbitrary and does not affect the most important determined value, which is the depth at which the detected object is located. 

**Figure 9:**
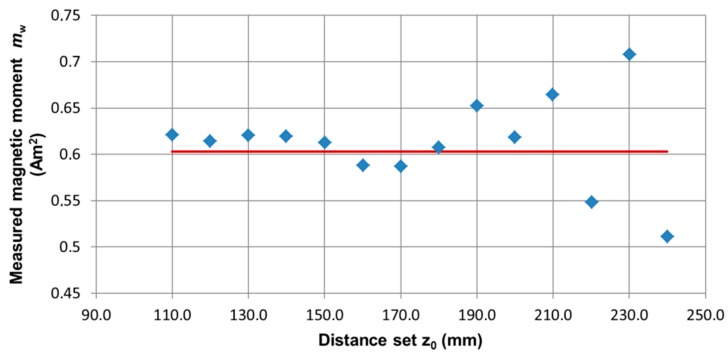
The dependence of the value of the determined magnetic moment of the object (*m_w_*) on the set distance *z_0_*. Position of the ME8 standard (*x*_0_, *y*_0_, *z*_0_) = (0, 0, 110:240) mm. Red line—*m* value of the object.

**Figure 10:**
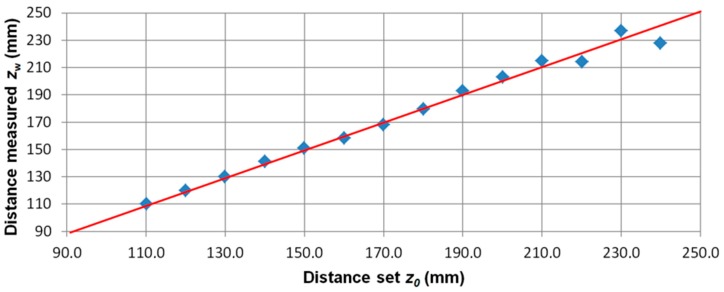
The dependence of the value of the determined distance from the object (*z_w_*) on the set distance *z*_0_. Position of the ME8 standard (*x*_0_, *y*_0_, *z*_0_) = (0, 0, 110:240) mm. Red line—*z*_0_ value of the object.

In the series of measurements with the change of coordinate *z_0_*, the standard was placed on a vertical linear table. The series of magnetovision measurements was made with the following parameters: coordinates *x*_0_ = 0, *y*_0_ = 0 mm, *z*_0_ changed in the range from 110 to 240 mm, angle *β* = 0 °, and angle *φ* = 0 °. Figure 9 shows the dependence of the value of the determined magnetic moment m of the object from the set distance *z*_0_, while in Figure 10, the dependence of the distance is measured from the given distance *z*_0_.

In laboratory conditions, the limitation was the noise generated by the equipment of the laboratory, at the level of 0.1 to 1 μT. In field conditions, outside of industrial zones, the background noise is significantly smaller, which makes it possible to detect objects using SQUID and fluxgate-type magnetometers at great depths (up to 5 m) [43,44]. 

The amplitude of the disturbance generated by the object is proportional to its magnetic moment and, in simplified form, inversely proportional to the distance cubed (*Z* axis). For this reason, a ten-fold increase in the distance of the measuring plane from a given object will result in a 10^3^-fold decrease in the amplitude of the disturbance. The results presented concern the distance from the object in the order of 0.2 m. In order to properly detect and locate such objects located at a distance of 2 m, a magnetometer with a 10^3^ times better resolution should be used, while ensuring a similar background noise level.

The measuring plane should cover all measurable field disturbances generated by the object sought. This can be ensured by magnetovision measurement over a larger area, and then measurement with a higher spatial resolution at the points of anomaly occurrence. In this case, the measuring plane should be a square with a side greater than or equal to the estimated distance from the object. If the magnetic sensor matrix is used, they should be firmly fixed relative to each other. If a scanning system with one triaxial magnetometer is used, maintaining the angular position is of paramount importance due to geomagnetic vector components affecting the measurement in the given sensor axis. 

The proper spatial resolution of the magnetovision measurement is required, but it may be unsymmetrical—a minimum of 10 measurement lines of 100 measurement points in each line, in the area of anomalies. Preliminary experimental research indicates no improvement in the location determination with further multiplication of measurement lines [45]. Decreasing the resolution results in inaccurate mapping of the anomaly shape and thus an increase in the error of determination of the object’s parameters.

In order to correctly determine all parameters of the object sought, i.e., the *x, y, z* coordinates and values of the magnetic moment vector, both the resolution value of the applied magnetometer and the low-frequency noise of the background field should be 10 times smaller than the amplitude of the disturbance generated by the searched object. The magnetic anomaly must therefore be significantly greater than the magnetic disturbances—chaotic fluctuations of the geomagnetic field, due to both natural and industrial factors [46,47]. 

## 4. Conclusions

The developed method of analysis of magnetovision measurements enables the remote determination of parameters of a hidden ferromagnetic object. The method allows simultaneous measurement of the position of the object in space (coordinates *x, y, z*) with respect to the measurement plane, as well as the value and direction of the resultant vector of the magnetic moment. During the experimental measurements, the distance z was determined with less uncertainty than the *x, y* coordinates; all coordinates were in the contour of the actual specimen. This allows us to unambiguously determine the location of a hidden ferromagnetic object. Automating the measurement reduces the possibility of human error, while its passive character increases safety when looking for dangerous objects.

The possibility to determine the value and direction of the magnetic moment, however, allows us to start work on identification of the hidden object. Available literature in this area indicates, for example, the existence of distinguished directions for unexploded ordnance (mortar shells and aerial bombs) that hit the ground [14]. The measurement of the magnetic moment value *m* of the object and its vector direction is performed with less uncertainty than with other recently described methods [28], while the measurement of the planar coordinates is slightly worse. The uncertainty would surely rise with the non-symmetric or elongated shape of the object, especially at close ranges. It was, however, not investigated in the presented research. 

For the first time, the detection and location method was verified using the certified magnetic moment standard, which enabled a quantitative assessment of the effectiveness, scope of applicability, and uncertainty of determining the individual parameters of the model. In recent literature, the magnetization/moment values obtained in the inversion procedure were not compared with its true values. Even more importantly, the presented Equation (1) and commercially available set of mathematical algorithms allow for fast inversion of the available datasets, with only the vertical field component *B_z_* values and measurement point coordinates needed. 

However, there are some disadvantages of the proposed approach in search of unexploded objects. The magnetic field anomalies of these objects in real conditions are in the 100 nT range. Magnetic disturbances can be of a similar magnitude. It would be a challenging task to create and use a large matrix of hundreds or thousands of rigidly bonded magnetic sensors in an urban environment or on rough terrain.

It would be also difficult to ensure the movement of magnetic sensors along specified trajectories with strict preservation of their orientation relative to the geomagnetic field. Additionally, most importantly, the surveyed area should first be thoroughly cleaned of ferromagnetic debris, common in every human settlement.

## Figures and Tables

**Table 1:** ME8 measurement results, with repeatability check.

	*m_w_* (Am^2^)	*z_w_* (mm)	*x_w_* (mm)	*y_w_* (mm)	*φ_w_* (°)	*β_w_* (°)	*c* (µT)
Mean value	0.575	165.7	35.2	-30.9	66.0	2.0	41.2
Standard deviation	0.020	1.6	1.9	1.4	1.5	0.8	0.2
Difference from the set value	0.059	0.7	5.2	-0.9	1.0	2.0	0.2
Relative error	9.7%	0.5%	14%	3%	1.5%	-	0.5%
